# A text message programme designed to modify patients’ illness and treatment beliefs improves self-reported adherence to asthma preventer

**Published:** 2012-06-15

**Authors:** John Weinman, Keith J Petrie, Kate Perry, Elizabeth Broadbent

**Affiliations:** Department of Psychological Medicine, Institute of Psychiatry, King’s College, UK; Faculty of Medical and Health Sciences, Department of Psychological Medicine, University of Auckland, New Zealand; Atlantis Healthcare, New Zealand; Faculty of Medical and Health Sciences, Department of Psychological Medicine, University of Auckland, New Zealand

**Keywords:** medicines, adherence, text message, asthma

## Abstract

**Background:**

Asthma is a common medical condition caused by chronic inflammation of the airways. Characteristic symptoms of the illness include attacks of shortness of breath, wheezing, tightness in the chest, and cough. Asthma is commonly treated by inhaled corticosteroids, which help to suppress inflammation of the airways and reduce the frequency of severe symptoms and attacks. This medication in the form of inhalers is known as preventer or controller medication and many patients also take short-acting bronchodilators to control acute symptoms (reliever medication). In order to provide therapeutic benefit, preventer medication needs to be taken regularly on a daily basis. However, non-adherence to preventer medication is a common problem in patients diagnosed with asthma and these results in the overuse of reliever medication, increased asthma symptoms, more frequent asthma attacks, and hospital admissions (Stern et al., 2006). Optimal adherence to inhaled corticosteroids requires patients to take their preventer medication on 80% or more occasions, as this is associated with greatest asthma control (Lasmar et al., 2009).

**Objective:**

While effective preventative medication is readily available for asthma, adherence is a major problem due to patients’ beliefs about their illness and medication. We investigated whether a text message programme targeted at changing patients’ illness and medication beliefs would improve adherence in young adult asthma patients.

**Methods:**

Two hundred and sixteen patients aged between 16 and 45 on asthma preventer medication were recruited from pamphlets dispensed with medication and e-mails sent to members of a targeted marketing website. Participants were randomized to receive individually tailored text messages based on their illness and medication beliefs over 18 weeks or no text messages. Illness and medication beliefs were assessed at baseline and at 18 weeks. Adherence rates were assessed by phone calls to participants at 6, 12, and 18 weeks and at 6 and 9 months.

**Results:**

At 18 weeks, the intervention group had increased their perceived necessity of preventer medication, increased their belief in the long-term nature of their asthma, and their perceived control over their asthma relative to control group (all p<0.05). The intervention group also significantly improved adherence over the follow-up period compared to the control group with a relative average increase in adherence over the follow-up period of 10% (p<0.001). The percentage taking over 80% of prescribed inhaler doses was 23.9% in the control group compared to 37.7% in the intervention group (p<0.05).

**Conclusion:**

A targeted text message programme increases adherence to asthma preventer inhaler and may be useful for other illnesses where adherence is a major issue.

